# Astaxanthin as a Modulator of Nrf2, NF-κB, and Their Crosstalk: Molecular Mechanisms and Possible Clinical Applications

**DOI:** 10.3390/molecules27020502

**Published:** 2022-01-14

**Authors:** Sergio Davinelli, Luciano Saso, Floriana D’Angeli, Vittorio Calabrese, Mariano Intrieri, Giovanni Scapagnini

**Affiliations:** 1 Department of Medicine and Health Sciences “V. Tiberio”, University of Molise, Via V. De Sanctis, s.n.c., 86100 Campobasso, Italy; intrieri@unimol.it (M.I.); giovanni.scapagnini@unimol.it (G.S.); 2 Department of Physiology and Pharmacology “Vittorio Erspamer”, Sapienza University, 00185 Rome, Italy; luciano.saso@uniroma1.it; 3 Department of Biomedical and Biotechnological Sciences, University of Catania, 95123 Catania, Italy; fdangeli@unict.it (F.D.); calabres@unict.it (V.C.)

**Keywords:** astaxanthin, oxidative stress, inflammation, Nrf2, NF-κB

## Abstract

Astaxanthin (AST) is a dietary xanthophyll predominantly found in marine organisms and seafood. Due to its unique molecular features, AST has an excellent antioxidant activity with a wide range of applications in the nutraceutical and pharmaceutical industries. In the past decade, mounting evidence has suggested a protective role for AST against a wide range of diseases where oxidative stress and inflammation participate in a self-perpetuating cycle. Here, we review the underlying molecular mechanisms by which AST regulates two relevant redox-sensitive transcription factors, such as nuclear factor erythroid 2-related factor 2 (Nrf2) and nuclear factor κB (NF-κB). Nrf2 is a cellular sensor of electrophilic stress that coordinates the expression of a battery of defensive genes encoding antioxidant proteins and detoxifying enzymes. Likewise, NF-κB acts as a mediator of cellular stress and induces the expression of various pro-inflammatory genes, including those encoding cytokines, chemokines, and adhesion molecules. The effects of AST on the crosstalk between these transcription factors have also been discussed. Besides this, we summarize the current clinical studies elucidating how AST may alleviate the etiopathogenesis of oxidative stress and inflammation.

## 1. Introduction

Astaxanthin (AST) is an oxygenated carotenoid belonging to a group of carotenoids called xanthophylls, which primarily includes lutein, zeaxanthin, β-cryptoxanthin, and canthaxanthin [1]. This compound occurs naturally in a wide variety of living organisms, including microalgae and plants, but it is especially found in the aquatic environment as a red-orange pigment common to many marine animals [2]. AST is synthesized by phytoplankton and marine bacteria and then passed on to fish through the food chain. However, fish grown in aquaculture (e.g., farmed salmonids) acquire the characteristic pink color to their flesh from AST feed supplement [3]. Although AST was initially employed as an animal food additive, when the first biological activities were reported and after an extensive review of its safety profile, the US Food and Drug Administration (FDA) approved the use of AST as a dietary supplement [4].

Recently, there has been an increasing scientific interest for AST in pharmaceutical and nutraceutical applications. AST has a number of configurational (stereo) isomers showing different physiological activities. The stereoisomers of AST include all-cis (3S, 3′S), cis-trans (3R, 3′S), and all-trans (3R, 3′R) [5,6]. Several sources of natural AST have been reported; however, the form obtained by the microalgae *Haematococcus pluvialis* is commonly used for human consumption. The unique molecular structure of AST enables it to attract free radicals or provide electrons and is responsible for its powerful antioxidant effect. Moreover, AST showed a greater ability to quench free radicals than other carotenoids, such as zeaxanthin and β-carotenoids [7,8,9].

A plethora of studies has demonstrated the mechanism by which continued oxidative stress leads to chronic inflammation, which, in turn, play a pivotal role in the pathophysiology of many chronic conditions such as cancer, diabetes, cardiovascular, and neurological diseases [10,11]. Extensive research has also revealed that AST exhibits multiple biological activities, including protection against oxidation of macromolecules and modulation of inflammatory responses. Notably, AST has emerged as potential key regulators of stress-sensitive transcription factors involved in antioxidant and anti-inflammatory mechanisms [12,13]. It has been reported that AST modulates nuclear factor erythroid 2-related factor (Nrf2), which binds to antioxidant response elements (ARE) in the promoter regions of most cytoprotective or detoxifying enzymes. Since Nrf2 regulates hundreds of genes that are involved in the response against oxidative stress, it is considered the central regulator of the maintenance of intracellular redox homeostasis [14,15]. Recent studies have also indicated that AST modulates the nuclear transcription factor-κB (NF-κB) signaling network, improving inflammation and oxidative stress in experimental models [16,17]. Several genes underlying inflammatory and stress responses have been shown to be transcriptionally regulated by NF-κB [18,19]. In addition, the complex interplay between Nrf2 and NF-κB has been investigated in relation to the risk of multiple age-related diseases. A growing body of research suggests that AST exhibits anti-aging effects, attenuating oxidative stress and inflammation through activation of Nrf2 and inhibition of NF-κB [20,21,22]. This review focuses on the biological activities and health benefits of AST, with a particular emphasis on the regulation of Nrf2 and NF-κB signaling pathways.

## 2. Overview on the Health Benefits of Astaxanthin

**Figure 1:**
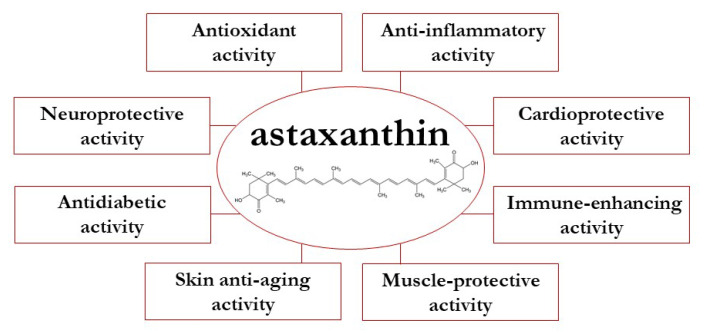
Bioactivity and health benefits of astaxanthin.

Redox homeostasis, an important component for the maintenance of normal physiological functions, is achieved by a well-controlled regulation between the formation and removal of reactive oxygen species (ROS) in the body system. An excess of oxidative molecules generated by a redox imbalance may damage cellular macromolecules, leading to a variety of detrimental effects associated with inflammation and disease development [23]. AST possesses the ability to modulate redox imbalance and inflammatory status and, therefore, it can be used as a potential tool in the clinical management of various chronic conditions [24,25,26]. Below, we give a summary of important biological activities mediated by AST (Figure 1).

### Biological Activity of Astaxanthin

Because of its peculiar chemical structure, especially the central polyene chain containing 13 conjugated double bonds, AST may act as a natural agent against oxidative damage by quenching singlet oxygen, scavenging free radicals, and preserving membrane structure through the inhibition of lipid peroxidation. For example, the AST polyene chain captures free radicals in the internal membrane of the cell, and, simultaneously, the terminal ring neutralizes reactive molecules on the surface of the membrane itself [27]. In addition, AST reduces ROS formation by increasing the expression of oxidative stress-responsive enzymes, such as superoxide dismutase (SOD), glutathione peroxidase (GPx), and catalase (CAT) [28]. The strong anti-oxidant properties of AST also play a crucial role in protecting biological systems from an uncontrolled inflammation associated with excessive levels of oxidative stress [29,30].

Accompanied by an increased ROS production, a wide variety of pro-inflammatory mediators are generated by M1 macrophages. In clinical and experimental studies, AST exhibits its antioxidant and anti-inflammatory actions by reducing the production of cytokines, such as interleukin-1β (IL-1β), interleukin-6 (IL-6), and tumor necrosis factor-α (TNF-α) [29,31]. However, AST also has the ability to reduce inflammation by inhibiting the cyclooxygenase-1 enzyme (COX-1) and nitric oxide (NO) [32,33]. In this context, AST has shown cardioprotective properties, reducing biomarkers of inflammation associated with cardiovascular damage and improving cardiac functions in mice. Moreover, it was observed that AST might limit the formation of atheroma by inhibiting the oxidation of low-density lipoproteins (LDL) and regulating the functions of macrophages involved in atherogenesis. Notably, AST also has antihypertensive effects through attenuation of the renin-angiotensin system and ROS-induced vasoconstriction [34,35,36].

Diabetes mellitus is characterized by decreased endogenous antioxidants, hyperglycemia, and impairment of the pancreatic β-cells. It has been reported that AST stimulates GPx activity, increases insulin levels, and improves glucose metabolism by modulating metabolic enzymes in diabetic animals. This effect protects pancreatic β-cells against glucose toxicity. Likewise, AST may modulate peroxisome proliferator-activated receptor gamma (PPAR-γ) with ameliorative effects on insulin resistance [37,38,39,40]. Besides its anti-diabetic properties, AST also exerts immune-enhancing effects. Various studies have shown that AST increases the immune response mediated by natural killer (NK) cells and T lymphocytes, as well as the production of immunoglobulin M (IgM), IgG, and IgA [29,41].

The anti-oxidant and anti-inflammatory effects of AST are also related to its neuroprotective activity. The level of non-enzymatic oxidative markers (e.g., malondialdehyde and NO), the activity of enzymatic antioxidants (e.g., SOD and CAT), and the expression of inflammatory cytokines (e.g., IL-1β, IL-6, and TNF-α) were positively modulated in different regions of the brain [42,43]. Accompanied by its free radical scavenging properties, AST also plays a crucial role against skin aging through activation of DNA repair mechanisms and inhibition of the matrix metalloproteinases (MMPs) that degrade collagen and elastin in the dermal skin layer [24]. Animal studies also revealed that AST decreased skeletal muscle injury induced by exercise [44].

## 3. Nrf2 and NF-κB as Key Players in the Crosstalk between Oxidative Stress and Inflammation

A sustained oxidative/inflammatory environment is an established risk factor for a wide variety of chronic diseases. A series of studies focused on elucidating the mechanisms involved in the onset and progression of chronic pathologies have concluded that oxidative stress results in increased inflammation. Therefore, these processes are part of a vicious cycle in which oxidative stress is both the cause and consequence of inflammation [45,46]. Eukaryotic cells have evolved a number of signal transduction pathways that orchestrate a rapid cellular response to a multitude of exogenous and endogenous stress-induced agents, including environmental pro-oxidants and inflammatory mediators. These pathways involve the stimulation of redox-sensitive transcription factors, such as Nrf2 and NF-κB. Their activation is critically involved in regulating the expression of an array of cellular genes associated with cytoprotective elements and inflammatory molecules [18,47].

In general, while Nrf2 confers cell protection in response to reactive species, inflammation, and xenobiotics, the activation of NF-κB occurs in response to similar stimuli but also controls the production of pro-inflammatory molecules [48,49]. Although the molecular mechanisms are not fully elucidated yet, current findings also suggest that Nrf2 and NF-κB affect each other to coordinate anti-oxidant and inflammatory responses. This interplay has also been investigated in relation to many degenerative and inflammatory diseases, and it could be a promising target against many pathological conditions [50].

### 3.1. Nrf2 Regulation in Oxidative Stress and Inflammation

Nrf2 is a ubiquitously expressed transcription factor, member of the Cap’n’collar (CNC) family, a subfamily of basic region-leucine zipper (bZIP) transcription factors. Nrf2 is composed of seven conserved functional domains (Neh1-7) regulating its transcriptional activity as well as its stability [51]. As a master regulator of the antioxidant response, Nrf2 is responsible for both constitutive and inducible expression of ARE-dependent genes, encoding cytoprotective proteins and detoxification enzymes. Most Nrf2 is sequestered by a repressor protein called Kelch-like ECH-associated protein1 (Keap1), a zinc metalloprotein localized primarily in the cytoplasm. Keap1 is rich in cysteine residues highly reactive and susceptible to electrophiles. Under basal/unstressed conditions, Keap1 promotes Nrf2 ubiquitination and proteasomal degradation in combination with Cullin 3-based E3 ligase (Cul3) complex. However, basal accumulation of Nrf2 in the nucleus is maintained to regulate the expression of ARE-dependent genes and guarantee cellular redox homeostasis [52,53].

In response to electrophilic or oxidative stress, Keap1 undergoes conformational changes and promotes the release of Nrf2, allowing its accumulation into the nucleus. The dissociation of Nrf2 from Keap1 can be also promoted through its phosphorylation by protein kinase C (PKC) and AMP-activated protein kinase (AMPK). In addition, the activation of Nrf2 can be regulated by glycogen synthase kinase-3β (GSK-3β), independent ofKeap1 activity [52,54,55]. Once inside the nucleus, Nrf2 heterodimerizes with one of the small Maf (musculoaponeurotic fibrosarcoma oncogene homolog) proteins and activates the ARE-dependent genes. The pool of genes within the ARE sequence encompasses approximately 600 genes encoding a series of redox balancing factors, detoxifying enzymes, and stress response proteins, such as heme oxygenase-1 (HO-1), nicotinamide adenine dinucleotide phosphate (NAD(P)H), NAD(P)H quinone oxidoreductase 1 (NQO1), glutathione S-transferase (GST), glutathione reductase (GR), carbonyl reductase (CR), glutamate-cysteine ligase catalytic subunit (GCLC), SOD, and GPx [14,56]. The transcriptional activation of these protective genes through Nrf2-ARE inducers derived from natural sources has gained enormous relevance in the development of new therapeutic strategies for several pathologies, including neurodegenerative diseases, diabetes, and cardiovascular diseases [15,57,58,59].

Besides its role as a master regulator of the antioxidant response, the Nrf2-ARE signaling pathway also regulates anti-inflammatory gene expression and inhibits the progression of inflammation. For example, an increase in the expression of HO-1, activated by Nrf2, leads to the inhibition of NF-κB, resulting in the inhibition of inflammatory mediators. However, numerous experimental studies in disease models have shown how activation of the Nrf2/HO-1 axis is accompanied by an attenuation of inflammatory reactions [47,60,61,62]. Furthermore, NQO1, GCLC, and HO-1 activated by Nrf2 may inhibit both cytokines and chemokines, including TNF-α, IL-6, IL-1β, monocyte chemoattractant protein-1 (MCP1), and macrophage inflammatory protein-2 (MIP2) [63,64]. It was also reported that Nrf2 suppresses the transcriptional activation of inflammatory genes without binding to ARE sequence. A study revealed that Nrf2 binds the promoter regions of pro-inflammatory cytokines (e.g., IL-6 and IL-1β) and inhibits the recruitment of RNA Pol II, which is, therefore, unable to activate the transcription of these genes [65].

### 3.2. NF-κB as Cellular Stress Response Pathway

The NF-κB is a widely expressed transcription factor involved in many cellular processes. In mammals, the family of NF-κB proteins includes RelA/p65, RelB, c-Rel, p50, and p52. These proteins are characterized by an N-terminal Rel homology domain (RHD) that establishes contact with DNA, promoting dimerization and binding to κB sites. In the canonical pathway, NF-κB is inhibited by IκB proteins. However, lipopolysaccharides (LPS), growth factors, antigen receptors, and pro-inflammatory cytokines can activate an IKK complex (IKKα and IKKβ), which phosphorylates IκB proteins, inducing their ubiquitination and subsequent proteasomal degradation. Hence, NF-κB translocates to the nucleus where, alone or in combination with other transcription factors, induces the transcription of target genes [66]. Conversely, the noncanonical pathway mediates the activation of the p52/RelB NF-κB complex. This NF-κB pathway relies on the inducible processing of p100, an NF-κB precursor, in response to a group of stimuli associated with ligands of a subset tumor necrosis factor receptor (TNFR) superfamily members [67].

A myriad of highly diverse genes has been demonstrated to be transcriptionally regulated by NF-κB, including immuno-receptors, growth factors, cell adhesion molecules, anti-apoptotic proteins, pathogens, chemotherapeutic agents, DNA damage, inflammatory mediators, and oxidative stress-related enzymes [68]. This is indicative of the pivotal role of NF-κB as a mediator of cellular stress. Remarkably, the NF-κB pathway exerts a double effect in many diseases. It is involved in the regulation of genes involved in inflammation and, likewise, mediates the expression of specific genes involved in the progression of the pathology [69]. Therefore, the NF-κB pathway may be a crucial therapeutic target in those conditions characterized by an elevated expression of this transcription factor and for which inflammation promotes organ damage.

However, several reports show that NF-κB is also involved in the regulation of oxidative stress response. For example, in a context-dependent manner, ROS can both activate and inhibit the NF-κB signaling pathway [70]. Notably, NF-κB has also been reported to regulate many antioxidant enzymes. One of the best-known targets is manganese superoxide dismutase (MnSOD) that is localized in the mitochondria. In particular, the levels of MnSOD are increased by TNFα through an NF-κB-dependent mechanism [71,72]. Other antioxidant enzymes induced by NF-κB include GST, NQO1, HO-1, and GPx [73]. Moreover, genes controlled by Nrf2, such as NQO1, GCLC, and glutamate-cysteine ligase modifier subunit (GCLM), also possess an NF-κB binding site [47]. Although there are conflicting results, several experimental studies have demonstrated that natural compounds or chemopreventive agents activate Nrf2 by inhibiting NF-κB and its regulated genes [57,74,75]. Conversely, ROS, LPS, oxidized LDL, and cigarette smoke have been shown to increase both Nrf2 and NF-κB activity [76]. This indicates crosstalk between Nrf2 and NF-κB signaling pathways, which may cooperate in a stimulus-specific manner.

## 4. Modulation of Nrf2 and NF-κB by Astaxanthin and Its Impact on Their Crosstalk

Accumulating evidence indicates that most carotenoids may be an important strategy for disease prevention and therapy [77,78]. These compounds can target a wide variety of signaling pathways, and their effects are often related to the modulation of Nrf2 and/or NF-κB pathways [79]. However, during the past 15 years, the literature has established a pivotal role of AST in regulating these transcription factors, thus exerting multiple health benefits [80].

### 4.1. Effects of Astaxanthin on Nrf2

Several experimental studies have reported that AST can protect the brain, heart, kidney, eyes, lungs, skin, and liver from oxidative stress, regulating Nrf2 and related factors [81,82,83,84,85,86]. The neuroprotective action of AST was associated with the activation of Nrf2 and its antioxidant enzymes in different experimental models of brain aging with an improvement of oxidative stress and mitochondrial dysfunction [87,88].

AST also alleviated brain damage in rats, upregulating Nrf2, HO-1, NADH, NQO-1, and GST and, accordingly, ameliorating cerebral oxidative stress [89]. Recently, it has been reported that AST protects against ochratoxin A (OTA)-induced myocardial injury through mitochondria-mediated apoptosis and activation of the Nrf2 pathway. This effect was accompanied by improved levels of cardiac and antioxidant enzymes [90].

The nephroprotective potential of AST was assessed in a rat model of streptozotocin-induced diabetes characterized by the accumulation of fibronectin in mesomeric glomerular cells challenged with high glucose (HG). The treatment promoted the transcriptional activity of Nrf2, as well as the expression of SOD1, NQO1, and HO-1, thus quenching the highest level of ROS and inhibiting HG-induced fibronectin, intercellular adhesion molecule 1 (ICAM1) and transforming growth factor β 1 (TGFβ1) expression. Therefore, these data suggested a nephroprotective effect of AST related to the activation of Nrf2-ARE signaling [91]. A high blood glucose level (i.e., hyperglycemia) induces oxidative stress and ROS generation in the retina, playing a role in the etiology of diabetic retinopathy. Different concentrations of AST on high glucose-cultured retinal cells attenuated apoptosis and induced phase II enzymes with the involvement of the Nrf2 pathway [92].

The treatment with AST increased Nrf2 expression in alveolar epithelial cells pre-treated with hydrogen peroxide. This increase was accompanied by elevated activity of SOD and CAT, indicating that AST may improve pulmonary oxidative stress by modulating Nrf2 [93]. Moreover, three in vivo studies showed that AST administration inhibited the development of chronic obstructive pulmonary disease (COPD) and acute lung injury through activation of Nrf2 and, accordingly, promoted HO-1 and inhibited Keap1 [85,94].

AST also plays an important role in the radioprotection and photoprotection of cells. Nrf2 and its principal targets, such as SOD, CAT, and GPx, were significantly upregulated in irradiated cells in the presence of AST. In addition, it has been reported that AST induced the expression of Nrf2 and HO-1 in dermal fibroblasts, increasing protection against UV [95,96].

The hepatoprotective activity of AST has been reported in an in vivo study. AST attenuated liver damage induced by doxorubicin, a chemotherapeutic drug. AST reduced ROS accumulation by downregulating the expression of Keap1 and activating Nrf2 together with SOD, CAT, and GPx. Moreover, AST improved the transaminases and reduced the damaged hepatocytes in mice [97]. AST also shows preclinical antiproliferative effects in various experimental models of cancer. For example, it has been observed that AST inhibits cancer growth in leukemia cells by modulating the Nrf2-ARE signaling pathway [98,99].

### 4.2. Astaxanthin and the Crosstalk between NF-κB and Nrf2

As mentioned, inflammation and oxidative stress are closely linked, and they widely contribute to multiple chronic diseases. For example, high ROS levels influence many aspects of cancer, such as sulfur-based metabolism, NADPH generation, and the activity of antioxidant transcription factors. The overexpression of NF-κB, which contributes to establishing an inflammatory microenvironment, was also widely observed in tumor samples and associated with cancer progression, metastasis, poor prognosis, and chemotherapy treatments [100,101].

Besides its role in regulating antioxidant genes via the Nrf2-ARE pathway, a large body of evidence indicates that AST treatment can inhibit NF-κB, which attenuates inflammation in both in vitro and in vivo studies [102,103]. A recent report in rodents indicated that the inhibitory effect of AST on NF-κB expression might be exerted via regulating the kinase subunits of the IKK complex. This effect was associated with decreased TNF-α and IL-6 secretion [104]. However, multiple studies have reported that AST can inhibit NF-κB activation. Suzuki et al. demonstrated that AST has a dose-dependent ocular anti-inflammatory effect through suppression of NO, TNF-α, and prostaglandin E2 (PGE2) production, which occurs by blocking the NF-κB transcription factor [105]. Similarly, another study demonstrated that AST reduced renal inflammation in a dose-related manner by regulating the NF-κB pathway and HO-1, ultimately preventing acute kidney injury [106]. Other authors have reported that AST inhibited NF-κB and Wnt/β-catenin signaling pathways in hepatocellular carcinoma cells and in a hamster model of oral cancer [103,107]. AST may also be a new candidate for the management of neuropathic pain by inhibiting neuroinflammation through the modulation of extracellular signal-regulated kinase (ERK)1/2, p38 mitogen-activated protein kinase (p38 MAPK), and NF-κB p65 [108]. AST also attenuated apoptosis after stretch injury in cultured astrocytes by reducing the expression of NF-κB-mediated pro-inflammatory factors [109]. Collectively, these findings emphasize how AST may exert anti-inflammatory effects through inhibition of the NF-κB transcription factors.

**Figure 2:**
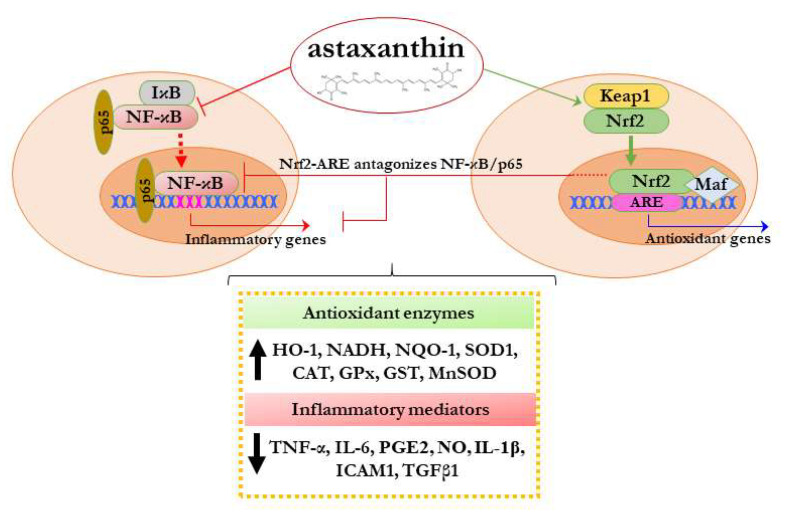
Schematic representation of the effect of astaxanthin on Nrf2 and NF-κB response pathways.

Although it is still under elucidation, recent studies have also demonstrated that AST may play a key role in modulating the complex interplay/crosstalk mechanism between Nrf2 and NF-κB pathways. Nrf2 activation has been related to its ability to antagonize NF-κB, suggesting that the induction of Nrf2 mediates anti-inflammatory responses (Figure 2) [110]. Moreover, in vitro and in vivo data have revealed that AST enhances Nrf2 activity and its antioxidant enzymes, inhibits pro-inflammatory mediators, and attenuates the NF-κB signaling network. Farruggia et al. have indeed shown that AST exerts its anti-inflammatory effect not only by inhibiting nuclear translocation of NF-κB p65 and decreasing the expression of IL-6 and IL-1β but also by reducing cellular ROS accumulation in Nrf2-dependent and -independent mechanisms [111].

Similarly, AST exerted protective effects against oxidative damage and inflammation induced by OTA in the lungs of mice. The treatment with AST increased the expression of Nrf2, HO-1, and MnSOD, whereas the expression of Keap1 and NF-κB significantly decreased [112]. Recently, Chen et al. investigated the underlying effects of AST treatment on aging animals. The anti-aging properties of AST were shown to be related to Nrf2 and NF-κB pathways and involved in cellular immunity. After AST treatment, Nrf2 expression was up-regulated, whereas Keap1, IL-1β, IL-6, and NF-κB p65 were significantly reduced. Interestingly, AST also elevated the levels of IL-2, immunoglobulin M (IgM), and immunoglobulin G (IgG), suggesting a novel mechanism by which AST could regulate cellular immunity and, eventually, attenuate immunosenescence [113].

## 5. Astaxanthin and Clinical Trials

Natural AST is a powerful antioxidant with translational implications for various physiological disorders. The bioavailability of AST in humans has been reported. After consuming a dose of 40 mg AST as a lipid-based formulation, the plasma concentration increased to ~190 μg/L, compared to subjects without supplementation [114]. Although there are no human studies assessing the effect of AST on Nrf2 and NF-κB transcription factors, promising clinical trials demonstrated its potential for the prevention or co-treatment of several human diseases, especially those related to oxidative stress, chronic inflammation, and aging.

Oxidative stress and neuroinflammation play a major role in brain aging. An increasing number of human studies have reported a potential neuroprotective effect of AST against cognitive impairment [115,116]. In a randomized, double-blind, placebo-controlled study, the effect of AST (12 mg/day) has been studied on the cognitive function of 96 elderly subjects. After 12 weeks of treatment, the results showed a significant improvement in cognitive performance, as the treated subjects demonstrated a faster response time in the CogHealth battery, a memory and thinking capability test [117].

Hyperlipidemia, lipid peroxidation, and pro-inflammatory factors have been associated with the etiology of many cardiovascular diseases, including atherosclerosis [118]. Three randomized, placebo-controlled trials reported that supplementation with AST (from 8 to 20 mg/day) might improve lipid profile (e.g., LDL, HDL, and triglyceride levels), decrease oxidation of fatty acids, and reduce biomarkers of oxidative stress, such as MDA and isoprostanes [119,120,121].

Emerging studies show that certain antioxidant phytochemicals may protect against various degenerative processes linked to visual impairment [122]. Currently, two clinical studies reported that ingestion of AST (6–12 mg/day) improved visual acuity and retinal blood flow [123,124]. There is also mounting evidence that AST possesses various clinical applications in the field of dermatology. A number of clinical trials indicated that AST might play a functional role in attenuating several oxidant events associated with skin aging, including DNA damage, reduced production of antioxidants, inflammatory responses, and the presence of matrix metalloproteinases (MMPs) [24]. In fact, many authors demonstrated that treatment with AST, orally or topically administered, reduced the levels of MDA, MMP-1, MMP-12, 8-hydroxy-2′-deoxyguanosine (8-OHdG), and pro-inflammatory mediators (e.g., IL-1α). These molecular changes were associated with an improvement of skin parameters, such as skin wrinkles, moisture content, age spots size, elasticity, sebum oil content, skin texture, and moisture content [24,125,126,127].

Some human intervention studies have also reported the potential benefits of AST on the immune system. For example, the results reported by Park et al. indicated that AST might enhance both cell-mediated and humoral immune responses, including T cell and B cell proliferation, natural killer (NK) cell cytotoxic activity, and IL-6 production [29].

Despite the small number of human trials, the clinical findings discussed in this paragraph suggest that AST may be a promising candidate for the prevention and co-treatment of several diseases associated with oxidative stress, inflammation, and aging.

## 6. Conclusions

AST is a multi-target compound that employs several mechanisms to exert its potential beneficial effects. Here, we reviewed experimental evidence showing that AST may confer cell protection against the detrimental effect of redox imbalance and chronic inflammation. This cytoprotective property is mainly mediated at the transcription level by modulating the complex biochemical network associated with Nrf2 and NF-κB signaling pathways. Therefore, in accordance with the findings discussed above, AST could be a promising agent against chronic disorders in which oxidative stress and inflammation are the main partners. However, most published studies have been performed in cells and animals using concentrations that are not achievable by humans.

Currently, no data demonstrate that Nrf2 and NF-κB are modulated by AST in humans. Despite this, preliminary clinical studies suggest that AST may be useful for the prevention and/or treatment of atherosclerosis, cognitive impairment, visual fatigue, and dermatological diseases. These human disorders are all associated with redox imbalance, inflammation, and aging. More well-designed clinical trials are needed to investigate whether AST may regulate Nrf2 and NF-κB in humans and protect against diseases characterized by excessive oxidative stress and inflammation.

## Data Availability

Not applicable.
